# Screws Fixation for Oblique Lateral Lumbar Interbody Fusion (OL-LIF): A Finite Element Study

**DOI:** 10.1155/2021/5542595

**Published:** 2021-05-15

**Authors:** Qinjie Ling, Huanliang Zhang, Erxing He

**Affiliations:** ^1^Dept of Spinal Surgery, The First Affiliated Hospital of Guangzhou Medical University, 151 Yanjiang West Road, Guangzhou, Guangdong, China 510120; ^2^MOE Key Laboratory of Disaster Forecast and Control in Engineering, Institute of Applied Mechanics, Jinan University, Guangzhou, Guangdong, China 510632

## Abstract

**Background:**

The combination of screw fixation and cage can provide stability in lumbar interbody fusion (LIF), which is an important technique to treat lumbar degeneration diseases. As the narrow surface cage is developed in oblique lateral lumbar interbody fusion (OL-LIF), screw fixation should be improved at the same time. We used the finite element (FE) method to investigate the biomechanics response by three different ways of screw fixation in OL-LIF.

**Methods:**

Using a validated FE model, OL-LIF with 3 different screw fixations was simulated, including percutaneous transverterbral screw (PTVS) fixation, percutaneous cortical bone trajectory screw (PCBTS) fixation, and percutaneous transpedical screw (PPS) fixation. Range of motion (ROM), vertebral body displacement, cage displacement, cage stress, cortical bone stress, and screw stress were compared.

**Results:**

ROM in FE models significantly decreased by 84-89% in flexion, 91-93% in extension, 78-89% in right and left lateral bending, and 73-82% in right and left axial rotation compared to the original model. The maximum displacement of the vertebral body and the cage in six motions except for the extension of model PTVS was the smallest among models. Meanwhile, the model PTVS had the higher stress of screw-rods system and also the lowest stress of cage. In all moments, the maximum stresses of the cages were lower than their yield stress.

**Conclusions:**

Three screw fixations can highly restrict the surgical functional spinal unit (FSU). PTVS provided the better stability than the other two screw fixations. It may be a good choice for OL-LIF.

## 1. Introduction

Screw fixation with cage in lumbar interbody fusion (LIF) is a key technique to treat lumbar disease degeneration. Wide surface cages were used in some minimally invasive LIF, such as oblique lumbar interbody fusion (O-LIF), direct/extreme lateral interbody fusion (DLIF/XLIF), and anterior lumbar interbody fusion (ALIF). However, injuries to the paraspinal nerves and vessels sometimes occur [1, 2]. Currently, an endoscopic technique through Kambin's triangle lumbar interbody fusion named as oblique lateral lumbar interbody fusion (OL-LIF) was introduced [3]. The safety and effectivity of the technique have been proved [4]. OL-LIF was a different form O-LIF, of which the approach was expanded via posterior retraction of the psoas for disc exposure [5]. Instead of the wide surface cage, the narrow surface cage was used by OL-LIF for inserting to the intervertebral space through an endoscopic working tunnel. Biomechanical evaluation showed that a narrow surface cage with 9 mm width was recommended in OL-LIF [6]. When a small cage was used, the demand of a screw-rods system for function spinal unit (FSU) stability was increased. The way of screw fixation in FSU was studied. One of them was transverterbral screws (TVS), also called transdiscal screws or transpedicular-transdiscal screws [7–9]. TVS was used to treat L5-S1 spondylolisthesis [7, 8, 10, 11] and thoracic interbody fusion [12]. Due to the multiple cortical bones across, FSU had more stability than that fixed by traditional transpedical screws (PS) and a high fusion rate was observed [12]. With the help of the SpineAssist miniature system [13] and the O-arm image guidance system [12], percutaneous transverterbral screws (PTVS) could be applied in the clinical setting. Similarly, cortical bone trajectory screw (CBTS) fixation was considered to have similar or even more stability than pedicle screws. Percutaneous cortical bone trajectory screws (PCBTS) have been applied well in LIF recently [14, 15]. However, which screw fixation has the most stability among PTVS, PCBTS, and percutaneous pedicle screws (PPS) have not been discussed.

Since 1973, Belytschko et al. [16] first developed a two-dimensional finite element (FE) model of the lumbar disc; FE analysis has become an effective method to study the biomechanics of the human spine. Compared with the in vitro cadaveric study, FE analysis had some advantages: lower economic cost, the easier to repeat the experiment, and better to predict the in vivo bone and spinal implant stress [17, 18]. Cage and screw fixation combination has been prove to achieve adequate vertebral stability in LIF [19, 20]. Recently, a novel narrow surface cage has been introduced to OL-LIF [6]. The result suggested that a 9 mm width cage was recommended in such minimally spinal surgery [6]. However, ways in screw fixation with the endoscopic cage for LIF have not been studied. The aim of this FE study was to evaluate three percutaneous screw fixations in biomechanics. The result can provide engineering evidence for the surgeon to improve the minimally invasive spinal surgery.

## 2. Materials and Methods

### 2.1. FE Model Development

**Figure 1:**
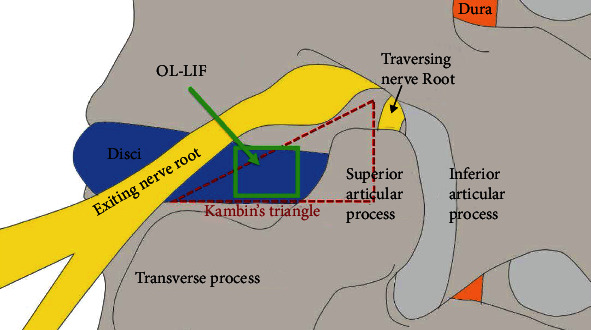
The surgical approach of endoscopic OL-LIF (direction of approach and surrounding anatomy: solid rectangle line was the endoscopic approach of OL-LIF and broken triangle line was the boundary of Kambin's Triangle. OL-LIF: oblique lateral lumbar interbody fusion).

**Figure 2:**
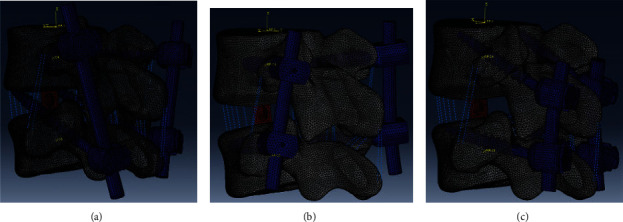
Endoscopic OL-LIF models with different screw fixation in Abaqus 6.14-4. (a) Model PTVS. (b) Model PCBTS. (c) Model PPS (PCBTS: percutaneous transverterbral screws fixation; PCBTS: percutaneous cortical bone trajectory screws; PPS: fixation and percutaneous transpedical screws).

The study was approved by the authors' affiliated institutions ethics committee. A FE software ABAQUS 6.14-4 (Dassault Systèmes, Vélizy-Villacoublay, France) was used to create a FE model of the L4-L5 functional spinal unit (FSU). The mesh sensitivity test and model validation have been done in previous study; the result of which is in a good agreement with other published experiments [6]. To simulate endoscopic OL-LIF (Figure 1), the disc of L4-L5 FSU and cartilage endplate were removed. Osteotomy was not needed in OL-LIF. Cortical bone and bony endplates were with 1 mm thickness. The thickness of facet joints in the contact area was 0.2 mm. The tangential behavior in contact was considered smooth, and normal behavior was described as a penalty algorithm. Seven major ligaments, the anterior longitudinal ligament (ALL), posterior longitudinal ligament (PLL), flaval ligament (FL), facet capsular ligament (CL), intertransverse ligament (ITL), interspinous ligament (ISL), and supraspinous ligament (SSL), were defined as axial connectors. The mechanical behaviors of all ligaments were described as nonlinear stress–strain curves. A peek cage with 9 mm width narrow surface and 11 mm height was inserted to the intervertebral space through Kambin's triangle by endoscopic approach [6]. Screws and rods were simulated as homogeneous linear elastic titanium (Ti-6Al-4V). Three different screw fixations (PTVS, PCBTS, and PPS) were assembled in the surgical models, respectively (Figure 2). The contact between screw heads and rods was defined as tied, where relative movement was forbidden. Additionally, contact between the screw and bone was simulated fully tied. The material properties of FE models can be seen in Tables 1 and 2.

### 2.2. Boundary and Loading Conditions

**Figure 3:**
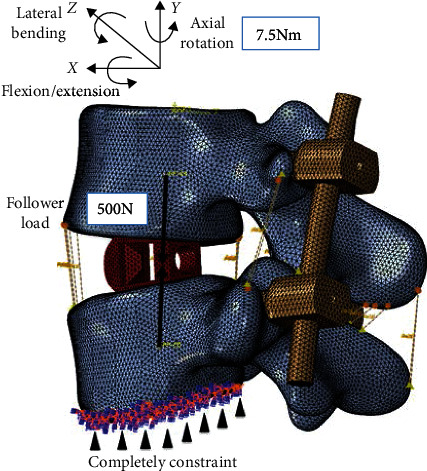
Loading and boundary conditions of the surgical model.

The inferior endplate of L5 was fully constrained. 500 N upper body weight in the lumbar spine was simulated by a compressive follower load with path through the centroids of two vertebral bodies (Figure 3). The moment of 7.5 Nm was applied on the superior endplate of L4 to test the motions of L4-L5 FSU in flexion, extension, lateral bending, and axial rotation (Figure 3).

## 3. Results

### 3.1. ROM and Displacement

**Figure 4:**
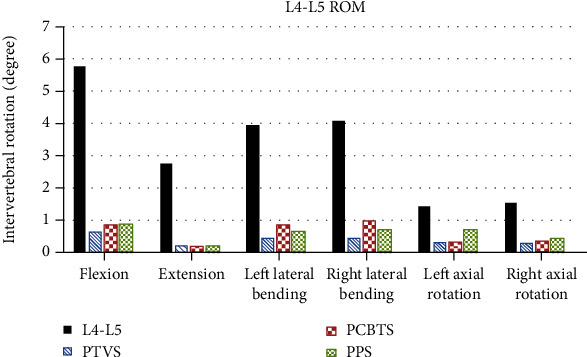
ROM of different FEA models (grey column: original model; blue column: PTVS; red column: PCBTS; green column: PPS).

Compared with the original model, ROMs of the three surgical models were significantly decreased by 84-89% in flexion, 91-93% in extension, 78-89% in right and left lateral bending, and 73-82% in right and left axial rotation (Figure 4). Model PTVS had the lowest ROM in five motions except in extension. In extension, the difference among surgical models was no more than 2%. The biggest difference happened in the right lateral bending between PTVS and PCBTS.

**Figure 5:**
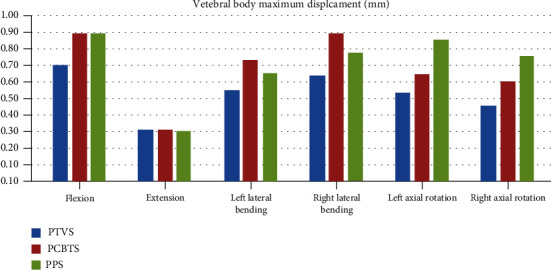
Vertebral body maximum displacement (mm) (blue column: PTVS; red column: PCBTS; green column: PPS).

**Figure 6:**
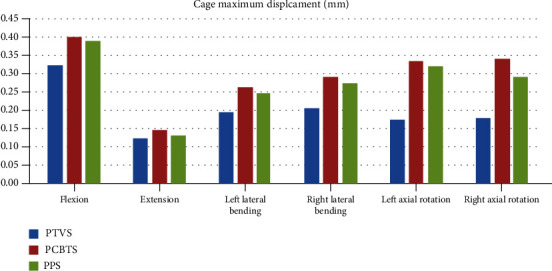
Cage maximum stress (MPa) (blue column: PTVS, red column: PCBTS, green column: PPS).

Among the models, the maximum displacement of the vertebral body and of the cage in model PTVS was the lowest in five moments, except that the extension was similar (Figures 5 and 6). Model PCBTS had the highest maximum displacement of the vertebral body in lateral bending, while the model PPS had in axial rotation (Figure 5). Model PCBTS also had the highest maximum displacement of the cage in all motions (Figure 6). The differences between model PCBTS and PPS were not obvious (Figure 6). Cages had the biggest displacement in flexion that model PTVS had 0.32 mm cage maximum displacement less than 0.40 mm for model PCBTS and 0.39 mm for model PPS (Figure 6).

### 3.2. The Maximum Equivalent von Mises Stress

**Figure 7:**
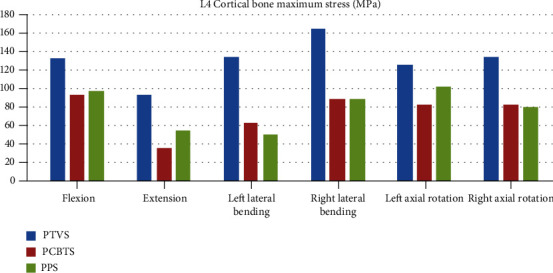
L4 cortical bone maximum stress (MPa) (blue column: PTVS, red column: PCBTS, green column: PPS).

**Figure 8:**
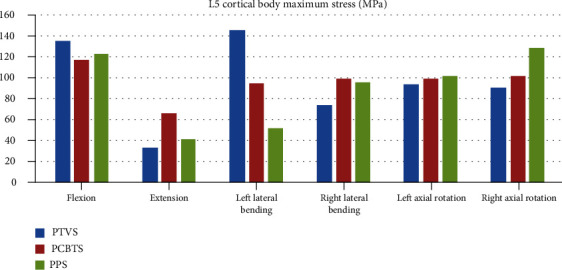
L5 cortical bone maximum stress (MPa) (blue column: PTVS; red column: PCBTS; green column: PPS).

**Figure 9:**
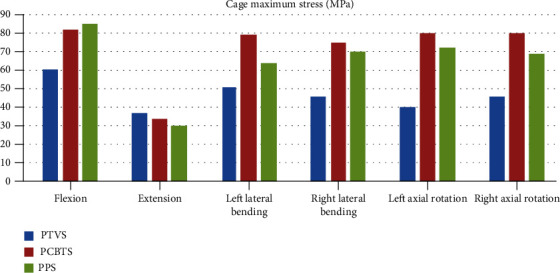
Cage maximum displacement (mm) (blue column: PTVS; red column: PCBTS; green column: PPS).

**Figure 10:**
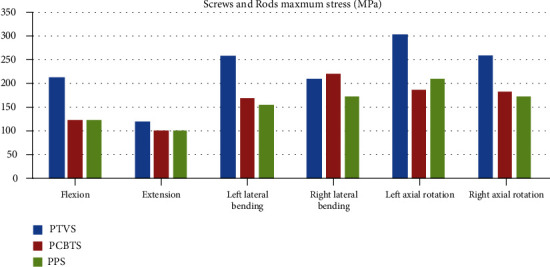
Screws and rods maximum stress (MPa) (blue column: PTVS; red column: PCBTS; green column: PPS).

The maximum stress of the L4 cortical bone in model PTVS was larger than the other two models in all moments (Figure 7). The largest stress was 132 MPa in PTVS in right lateral bending (Figure 7). The difference between PCBTS and PPS was not obvious. PCBTS had the lowest stress 36 MPa in extension among the models (Figure 7). The maximum stress of the L5 cortical bone was the largest in flexion and in left lateral bending in model PTVS, while it was the largest in extension and in right lateral bending in model PCBTS and was largest in left/right axial rotation in model PPS (Figure 8). In flexion, cage suffered the highest stress, which in PTVS was 57 MPa, in PCBTS was 82 MPa, and in PPS was 85 MPa. Among the models, the cage maximum stress of model PTVS was the smallest in five moments except for extension (Figure 9). Screw and rod maximum stress of model PTVS was the largest among the models in all motions; those were obvious in flexion, left lateral bending, and left/right axial rotation. In model PTVS, screw fixation maximum stress was 307 MPa, larger than 224 MPa for model PCBTS and 209 MPa for model PPS (Figure 10).

## 4. Discussion

Stability of the lumbar spine refers to the ability of the lumbar to cope with the physiological load of daily life, which is mainly maintained by the lumbar intervertebral disc, facet joint, intervertebral ligaments, and muscles. LIF with screw-rods system combined with cage can provide a strong stability for the lumbar segment and create an environment for the transplanted bone tissue for solid fusion. The way of screw fixation and the size of the cage both affect the stability of postoperative FSU. As the minimally invasive spinal surgery developed, the size of the cage got smaller than before to match the endoscopic working tunnel. When the cage changed, the screws should be enhanced. PTVS, PCBTS, and PPS have actually been put on the professional treatment of spinal disease. This study utilized a finite element method to assess the biomechanics of three screw fixations in endoscopic LIF. The result provided engineering evidence to the surgeon for reference in the clinical setting.

The ROM and the maximum displacement of the FE model can directly reflect the stability of the postoperative model. The smaller the ROM and displacement of FE model were, the greater the limit of the model activity, and the better the stability of the postoperative model. A solid stability of the postoperative FSU is good for intervertebral bone fusion because the micromotion of the cage in the intervertebral space can hinder bone fusion [21, 22]. In this study, the bottom of L5 was completely constricted and the maximum displacement of the model occurred in the anterior upper side of the L4 vertebral body. The displacement of the L4 vertebral body can mirror the ROM of the surgical model. A smaller L4 displacement resulted in lower ROM of FSU and higher stability of model. Displacement of L4 in six motions except extension of model PTVS was smaller than that of model PCBTS and model PPS (Figure 5). Similarly, the displacement of cage in six motions except the extension of the cage in model PTVS was smallest among the three models (Figure 6). Therefore, the model PTVS had the best stability among the models. The difference of extension among models was small, which agreed with the clinical setting because of the obstruction of the upper and lower articular process, lamina, spinous process, and ligaments. PTVS can effectively restrict the movement of the vertebral body by traversing multiple layers of the cortical bone (posterior pedicle cortex of L5, superior cortex of L5, and inferior cortex of L4), strongly fixing the anterior, middle, and posterior columns of L4-L5.

In LIF, after discectomy, the stability of FSU was mainly maintained by the cage and screw-rods system in addition to the facet joint and posterior osseous ligament complex. The stress of the cage was inversely proportional to that of the screw-rods system. The smaller the stress of cage, the greater the stress experienced by the screw-rods system, especially in minimally invasive LIF. In flexion, PTVS prevented L4 from moving downward and shared the pressure caused by L4, so the strain and stress of the cage were reduced (Figures 6 and 9). On the one hand, the proper pressure of the cage could prevent the movement of the implanted bone tissue and cage, which was good for interveterbral bone fusion. On the other hand, the cage maximum stress was no more than the yield stress 95 MPa [23] and that it would not cause fatigue rupture due to excessive stress. According to Wolff's law, the bone tissue has the ability to adapt to the mechanical environment. Proper stress can stimulate the growth of the bone tissue.

The contact area between the PTVS and the cortical bone of the vertebral body was small, so it was easy to cause local contact stress concentration. However, the stress concentration was not obvious from the result of calculation. The stress distribution of PTVS is more even than that of the other two fixations. The maximum stress of the PTVS was distributed more evenly in the screw body, while those of the PCBTS and PPS were distributed at the junction between the screw body and the screw head. PTVS has a longer body than PCBTS and PPS. Longer screw in the vertebral body could provide a better fatigue test, which could a bear better circulating load [24]. Using the large diameter screw may provide a better fatigue test, and the fusion cage could be considered omitted.

From the discussion above, it can be found that PTVS had the better stability than the other two screw fixations. Crossing the multiple layers of the cortical bone, PTVS effectively constrained the FSU sharing load stress with cage. With PTVS, the narrow surface cage in LIF was hardly destroyed. In the future, when the material of the screw improves and the fatigue test was passed, maybe there is no need for using the cage in LIF.

### 4.1. Limitations of This Study

The model of this study selected a FSU and did not simulate the whole lumbar vertebral model. The effect on the postoperative adjacent lumbar intervertebral disc was not considered. Cyclic load on the lumbar was not taken into account. Some components were simplified, e.g., the ligaments were modeled as an axial connector. The bone graft and postoperative residual annular fibrous were not constructed in the models because until bone fusion neither of these structures could provide immediate mechanical support after surgery. The cage was also simplified to remove the serrated surface. In our future study, the fatigue failure test of screws and cage will be investigated.

## 5. Conclusion

This study used a finite element method to develop three surgical models to simulate OL-LIF. The result showed PTVS restricted the model most displacement among models. Postoperative PTVS could provide strong stability for FSU immediately. PTVS combined with a narrow surface cage may be a good choice for OL-LIF.

## Figures and Tables

**Table 1:** Parameters of the various tissues of the lumbar spine. [5].

Tissues	Modulus (MPa)	Poisson's ratio	Element type	Thickness
Cortical bone	12000	0.3	Shell	1 mm
Cancellous bone	100	0.2	Solid	/
Bony endplate	12000	0.3	Shell	1 mm
Cartilaginous endplate	23.8	0.4	Shell	0.8 mm
Facet	35	0.4	Shell	0.2 mm
Titanium (Ti-6Al-4V)	110000	0.3	Solid	/
PEEK (polyetheretherketone)	3700	0.3	Solid	/

**Table 2:** Property of seven ligaments of the lumbar spine. [5].

Ligament	Strain: *ε* (%)	Stiffness: *k* (N/mm)	*ε* (%)	*k* (N/mm)	*ε* (%)	*k* (N/mm)	*ε* (%)	*k* (N/mm)
ALL	*ε* < 0	*k* = 0	0 < *ε* < 12.2	347	12.2 < *ε* < 20.3	787	20.3 < *ε*	1864
PLL			0 < *ε* < 11.1	29.5	11.1 < *ε* < 23	61.7	23 < *ε*	236
CL			0 < *ε* < 25	36	25 < *ε* < 30	159	30 < *ε*	384
ITL			0 < *ε* < 18.2	0.3	18.2 < *ε* < 23.3	1.8	23.3 < *ε*	10.7
FL			0 < *ε* < 5.9	7.7	5.9 < *ε* < 49	9.6	49 < *ε*	58.2
SSL			0 < *ε* < 20	2.5	20 < *ε* < 25	5.3	25 < *ε*	34
ISL			0 < *ε* < 20	1.4	13.9 < *ε* < 20	1.5	20 < *ε*	14.7

## Data Availability

The processed data required to reproduce these findings cannot be shared at this time as the data also forms part of an ongoing study.
